# Whether partial colectomy is oncologically safe for patients with transverse colon cancer: a large population-based study

**DOI:** 10.18632/oncotarget.21275

**Published:** 2017-09-26

**Authors:** Xu Guan, Zhixun Zhao, Ming Yang, Haipeng Chen, Wei Chen, Zheng Liu, Zheng Jiang, Yinggang Chen, Guiyu Wang, Xishan Wang

**Affiliations:** ^1^ Department of Colorectal Surgery, National Cancer Center/Cancer Hospital, Chinese Academy of Medical Sciences and Peking Union Medical College, Beijing, China; ^2^ Department of Colorectal Surgery, The Second Affiliated Hospital of Harbin Medical University, Harbin, China; ^3^ Department of Surgical Oncology, The First Affiliated Clinical Hospital of Qiqihaer Medical University, Qiqihaer, China; ^4^ Follow Up Center, The Second Affiliated Hospital of Harbin Medical University, Harbin, China

**Keywords:** hemicolectomy, colectomy, transverse colon cancer, survival

## Abstract

Due to special tumor location and technical difficulty of transverse colon cancer (TCC), partial colectomy (PC) is being widely applied in selected TCC patients, instead of extended hemicolectomy (HC). However, the oncological safety of this less aggressive surgical approach is not well studied. Here, we identified 10344 TCC patients from Surveillance, Epidemiology, and End-Results (SEER) database. The surgical treatment for those patients included PC and HC. Firstly, we compared lymph nodes evaluations between patients treated with HC and PC, including median number of nodes, the rate of nodes ≥ 12 and the rate of node positivity. Then, 5-year cancer specific survival (CSS) was obtained. Kaplan-Meier methods and Cox regression models were performed to assess the correlations between prognostic factors and long-term survival. Despite of less node examined by PC, the rate of node positivity was equal between PC and HC, suggesting node retrieval under PC was adequate to tumor stage. In addition, the 5-year CSS for patients who underwent PC were 67.5%, which was similar to patients who received HC (66.5%). The result after propensity score matching also confirmed the equivalent survival outcome between HC and PC. However, subgroup analyses showed that patients with tumor size ≥ 5 cm could not obtain survival benefit from PC. Furthermore, surgical approach was not considered as independent prognostic factor for TCC patients. Therefore, although PC is a less aggressive surgical approach, it should be a safe and feasible option for selected TCC patients.

## INTRODUCTION

The transverse colon cancer (TCC) accounts for about 10% of all colorectal cancer [1]. Surgical treatment is still play the leading role in the curative treatment for the vast majority of colon cancer patients [2–4]. The surgical approach and the extent of lymph node dissection for CC depend on tumor location, extent of lymphatic spread and oncologic outcome. The central tumor location of TCC presented difficult surgical choices in determining appropriate surgical procedure and extent of lymphadenectomy, reconstruction of intestinal continuity, as well as technical difficulties regarding identification, ligation, and lymph node dissection around the middle colic vessels [5]. Furthermore, due to TCC patients were excluded from prior randomized controlled trials, reasonable surgical procedure should be cautiously weighed for this group of patients [6, 7].

Currently, the surgical treatment for TCC patients is still lack of “golden standard”, the oncological outcomes of surgical treatment for TCC is also unclear, which posed a great challenge for surgeons to decide appropriate surgical approach for TCC patients. The main surgical approaches for TCC patients include less aggressive partial colectomy (PC) and extended hemicolectomy (HC). It is well acknowledged that HC is associated with larger extent of colon resection and more lymph nodes examined, which lead to more technical difficulties in surgical procedure. Therefore, surgeons alternatively perform the less aggressive PC in selected TCC patients. However, regardless of difficulties on surgical technique, the major controversy about PC for TCC lies on whether or not it is feasible to perform sufficient extent of lymph node dissection and equivalent survival outcome compared with extended HC.

The aims of this study were to establish for the first time to compare the oncological outcomes between PC and extended HC for TCC patients based on a large-scale national cohort study. Firstly, we compared lymph nodes evaluations including the median number of lymph node, the rate of nodes ≥ 12 and the rate of node positivity between PC and HC. Secondly, we compared the long-term outcomes of TCC patients who underwent PC and HC by propensity score matching (PSM) analysis. Thirdly, we divided patients into 17 subgroups based on different demographic and clinicopathological characteristics to further confirm the prognostic value between two surgical procedures.

## RESULTS

### Patient characteristics

A total of 10344 eligible TCC patients were collected during 10 years period, which included 4431 patients who underwent PC and 5913 patients who underwent HC. Of the cohort, the proportion of female patients was 52.5%, white patients was 80.3%, accounting for the majority of patients collected. In PC group, 24.5% of patients were in stage I, 41.1% in stage II and 34.4% in stage III. 55.4% of patients were aged ≥ 70 years; this proportion was decreased to 52.1% in HC group. Tumor in T3/T4 stage accounted for 71.7% in PC group, which was obviously lower than HC group (78.3%). Patients with tumor size ≥ 5 cm accounted for 34.0% in PC group, this proportion increased to 40.8% in HC group. The proportions of patient with adenocarcinoma were 89.1% and 87.9% in PC and HC group separately. The detailed information was listed in Table 1.

**Table 1:** Characteristics among transverse colon cancer patients

Characteristics	Partial colectomy *N* = 4431		Hemicolectomy *N* = 5913		*P*
**Gender**					0.015
**Male**	2042	46.1%	2867	48.5%	
**Female**	2389	53.9%	3046	51.5%	
**Age (Years)**					0.001
**< 70**	1975	44.6%	2834	47.9%	
**≥ 70**	2456	55.4%	3079	52.1%	
**Race**					0.029
**Black**	501	11.3%	742	12.5%	
**White**	3564	80.4%	4746	80.3%	
**Others**	366	8.3%	425	7.2%	
**AJCC TNM Stage**					< 0.001
**Stage I**	1086	24.5%	1124	19.0%	
**Stage II**	1820	41.1%	2755	46.6%	
**Stage III**	1525	34.4%	2034	34.4%	
**AJCC T stage**					< 0.001
**T1/T2**	1252	28.3%	1285	21.7%	
**T3/T4**	3179	71.7%	4628	78.3%	
**AJCC N stage**					0.985
**N0**	2906	65.6%	3879	65.6%	
**N1/ N2**	1525	34.4%	2034	34.4%	
**Grade**					< 0.001
**Grade I/II**	3626	81.8%	4598	77.8%	
**Grade III/IV**	805	18.2%	1315	22.2%	
**Histology**					0.070
**Adenocarcinoma**	3947	89.1%	5199	87.9%	
**Mucous Tumor**	484	10.9%	714	12.1%	
**Tumor size (cm)**					< 0.001
**0–5**	2925	66.0%	3503	59.2%	
**≥ 5**	1506	34.0%	2410	40.8%	
**Year of diagnosis**					0.356
**2004–2008**	2246	50.7%	2943	49.8%	
**2009–2013**	2185	49.3%	2970	50.2%	

### Comparisons of lymph node evaluation between HC and PC group

**Figure 1:**
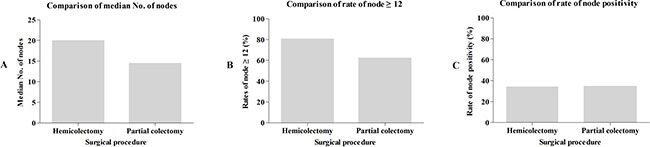
(**A**) The comparison of median number of lymph nodes examined between HC and PC. (**B**) The comparison of the rate of ≥ 12 lymph nodes examined between HC and PC. (**C**) The comparison of the rate of node positivity between HC and PC. HC: hemicolectomy; PC: partial colectomy.

To compare the differences of lymph node examination between PC and HC, we evaluated the median number of node, the rate of node ≥ 12 and the rate of node positivity between HC and PC. Patients in HC group had a median number of 19.9 nodes, which was obviously higher than patients who underwent PC (14.3) (A). A harvest of ≥ 12 nodes was considered as adequate nodal evaluation, < 12 nodes was defined as poor node retrieval. Accordingly, we compared the rate of node ≥ 12. The results showed that the rate of ≥ 12 nodes for patients who underwent HC was 80.3%, which was significantly higher than patients who underwent PC (62.0%) (Figure 1B). Although increased number of nodes examined by HC, the rate of node positivity for patients treated with HC was 34.4%, which was same to patients treated with PC (34.4%) (Figure 1C). This finding indicated that less aggressive PC for TCC do not lead to decreased rate of node positivity. Therefore, this result implies that node retrieval under PC is enough to determine tumor stage, and PC should be considered as adequate surgery in selected TCC patients.

### Survival comparison between HC and PC group

**Figure 2:**
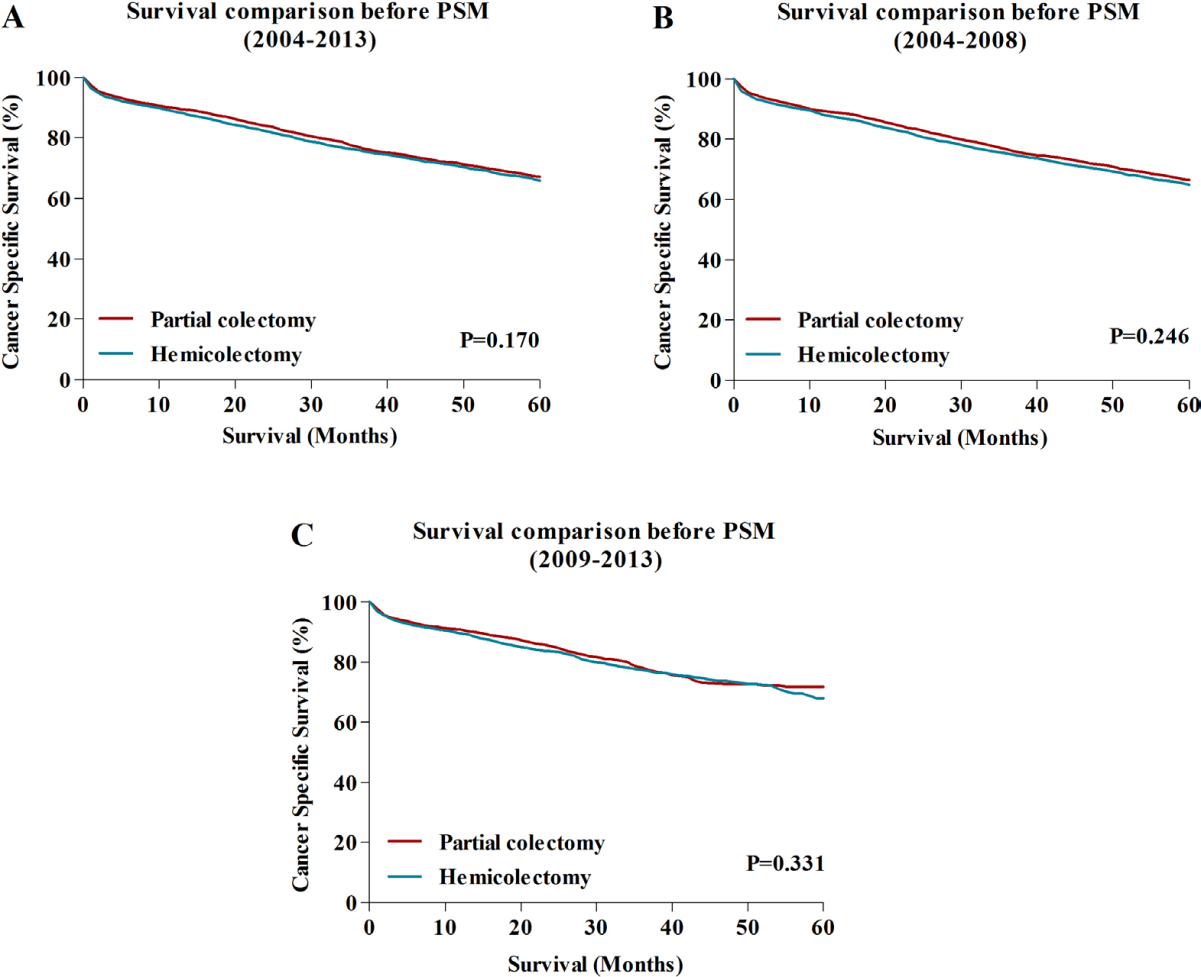
(**A**) The comparison of 5-year CCS between HC and PC during 2004–2013 before PSM analysis. (**B**) The comparison of 5-year CCS between HC and PC during 2004-2008 before PSM analysis. (**C**) The comparison of 5-year CCS between HC and PC during 2009–2013 before PSM analysis. HC: hemicolectomy; PC: partial colectomy.

With the aim of estimating whether the less aggressive PC could influence the long-term survival benefit, we compared the 5-year CSS between patients in HC and PC group. The results showed that the 5-year CSS for patients who underwent HC were 66.5%, which were similar to patients who received PC (67.5%), the survival difference has no statistical significance (*P* = 0.170) (Figure 2A). Furthermore, to avoid potential influence of advanced surgical techniques and devices on survival, we stratified patients to examine the relationship between surgical approach and survival in two different periods separately, the year of TCC diagnosis during 2004–2008 and 2009–2013. The results indicated that the survival in both periods of 2004–2008 and 2009–2013 could not be reduced by this less aggressive surgical resection (Figure 2B and 2C).

**Figure 3:**
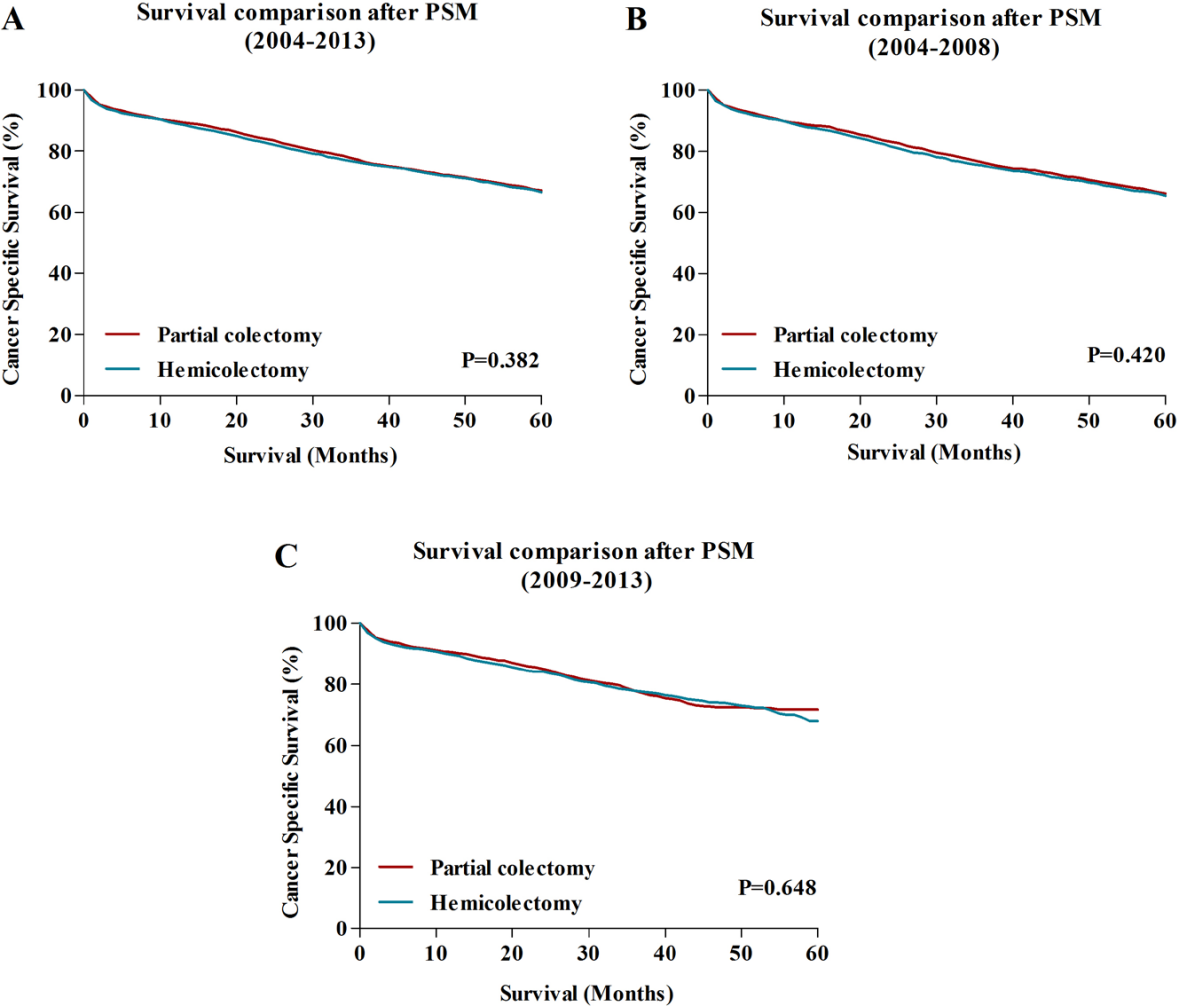
(**A**) The comparison of 5-year CCS between HC and PC during 2004–2013 after PSM analysis. (**B**) The comparison of 5-year CCS between HC and PC during 2004–2008 after PSM analysis. (**C**) The comparison of 5-year CCS between HC and PC during 2009-2013 after PSM analysis. HC: hemicolectomy; PC: partial colectomy.

Then, the PSM analysis was performed to reduce possible bias between HC and PC group. After matching, there were totally 8602 patients left, with 1:1 ratio in HC group and PC group. No difference was observed between two groups in terms of gender, age, AJCC TNM stage, AJCC T stage, AJCC N stage, grade, histology, tumor size and year of diagnosis, with *P* > 0.05. Table 2 showed that all characteristics were well balanced after PSM. Then, the 5-year CSS was compared between patients in HC and PC group. The results were similar to the primary survival comparisons before PSM. The 5-year CSS for patients treated with HC was 66.5%, which was similar to those received PC (67.0%) (*P* = 0.382) (A). The stratified analyses during the period of 2004-2008 and 2009–2013 also confirmed this result (Figure 3B and 3C).

**Table 2:** Characteristics among transverse colon cancer patients after matching

Characteristics	Partial colectomy *N* = 4301		Hemicolectomy *N* = 4301		*P*
**Gender**					1
**Male**	2000	46.5%	2000	46.5%	
**Female**	2301	53.5%	2301	53.5%	
**Age (Years)**					1
**< 70**	1933	44.9%	1933	44.9%	
**≥ 70**	2368	55.1%	2368	55.1%	
**Race**					0.143
**Black**	483	11.2%	526	12.2%	
**White**	3465	80.6%	3459	80.4%	
**Others**	353	8.2%	316	7.4%	
**AJCC TNM Stage**					1
**Stage I**	1011	23.5%	1011	23.5%	
**Stage II**	1818	42.3%	1818	42.3%	
**Stage III**	1472	34.2%	1472	34.2%	
**AJCC T stage**					1
**T1/T2**	1144	26.6%	1144	26.6%	
**T3/T4**	3157	73.4%	3157	73.4%	
**AJCC N stage**					1
**N0**	2829	65.8%	2829	65.8%	
**N1/ N2**	1472	34.2%	1472	34.2%	
**Grade**					1
**Grade I/II**	3517	81.8%	3517	81.8%	
**Grade III/IV**	784	18.2%	784	18.2%	
**Histology**					1
**Adenocarcinoma**	3853	89.6%	3853	89.6%	
**Mucous Tumor**	448	10.4%	448	10.4%	
**Tumor size (cm)**					1
**0–5**	2819	65.5%	2819	65.5%	
**≥ 5**	1482	34.5%	1482	34.5%	
**Year of diagnosis**					0.137
**2004–2008**	2187	50.8%	2118	49.2%	
**2009–2013**	2114	49.2%	2183	50.8%	

### 17 subgroup analyses

**Figure 4:**
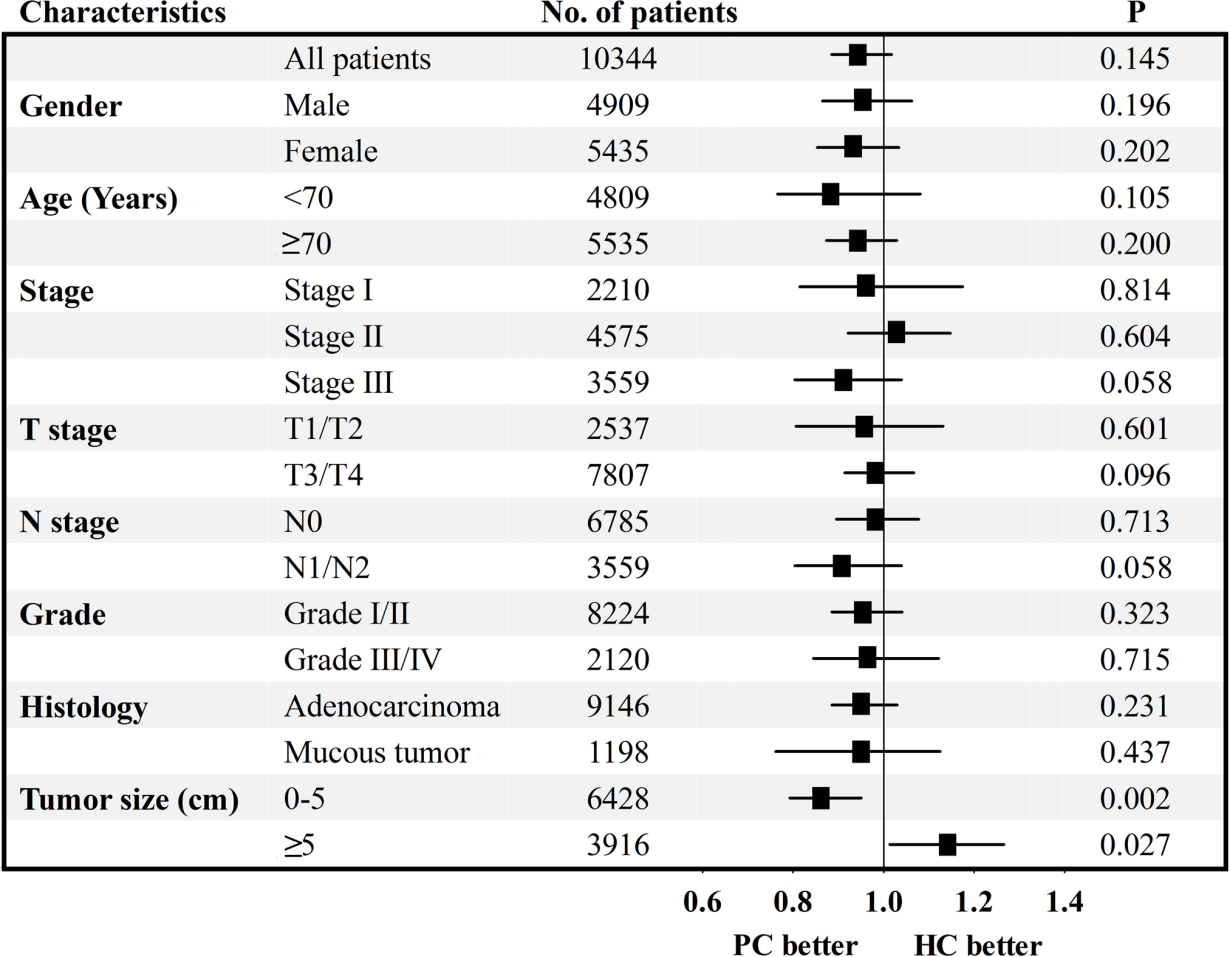
Survival comparisons between PC and HC in 17 subgroup analyses HC: hemicolectomy; PC: partial colectomy.

To further observe the prognostic value between two surgical approaches, we carried out the subgroup analysis, and divided patients into 17 subgroups based different demographic and clinicopathological characteristics. Cox's regression models were separately used to calculate HR and 95% CIs in each subgroup (Figure 4). The results showed that patients who underwent HC could not obtain much more survival benefits than patients treated with PC. The influence of surgical approach with respect to CSS was homogeneous in 15 subgroups with *P* > 0.05. However, for TCC patients with tumor size ≥ 5 cm, they could obtain survival benefit from extended HC. For patients with tumor size < 5 cm, they had better long-term outcome from less aggressive PC. Therefore, this finding sufficiently established that TCC patients treated with PC showed similar long-term survival outcome to patients who underwent HC, except for patient with large tumor size.

### Identifying adverse prognosis factors for TCC patients

With the aim of identifying the factors that influenced long-term survival of TCC patients, univariate and multivariate Cox regression analyses were performed to determine prognostic factors (Table 3). The results suggested that TCC patients who underwent PC were not considered as independent adverse prognostic factor for CSS. However, other characteristics including aged ≥ 70 years, black, stage T3/T4, stage N1/N2, grade III/IV and tumor size ≥ 5 cm were all identified as independent adverse prognostic factors.

**Table 3:** Univariate and multivariate analyses for transverse colon cancer patients

Characteristic		Univariate analysis		Multivariate analysis	
		HR [95% CI]	*P*	HR [95% CI]	*P*
**Gender**	Female	1	0.403	1	0.612
	Male	0.971 [0.906–1.040]		0.921 [0.891–1.033]	
**Age (Years)**	< 70	1	< 0.001	1	< 0.001
	≥ 70	2.901 [2.683–3.138]		3.112 [2.874–3.370]	
**Race**	White	1	< 0.001	1	< 0.001
	Black	1.056 [0.951–1.172]		1.243 [1.118–1.381]	
	Others	0.660 [0.566–0.770]		0.682 [0.584–0.796]	
**AJCC TNM Stage**	Stage I	1	< 0.001	1	0.094
	Stage II	1.514 [1.362–1.683]		0.771 [0.600–0.992]	
	Stage III	2.244 [2.020–2.493]		0.645 [0.370–1.125]	
**AJCC T stage**	T1/T2	1	< 0.001	1	< 0.001
	T3/T4	1.799 [1.640–1.974]		1.837 [1.460–2.312]	
**AJCC N stage**	N01	1	< 0.001	1	0.007
	N1/N2	1.692 [1.578–1.815]		2.011 [1.209–3.345]	
**Grade**	Grade I/II	1	< 0.001	1	< 0.001
	Grade III/IV	1.482 [1.369–1.605]		1.211 [1.115–1.315]	
**Histology**	Adenocarcinoma	1	0.021	1	0.918
	Mucous Tumor	1.127 [1.018–1.248]		1.005 [0.907–1.115]	
**Tumor size (cm)**	0–5	1	< 0.001	1	0.001
	≥ 5	1.265 [1.179–1.357]		1.136 [1.055–1.222]	
**Surgical procedure**	Partial colectomy	1	0.146	1	0.335
	Hemicolectomy	1.053 [0.982–1.130]		1.035 [0.965–1.111]	

## DISCUSSION

Currently, none of study paid attention to the influence of surgical approaches on oncological outcomes among TCC patients. Furthermore, the TCC patients are not included in high quality studies, which have resulted in a lack of evidence-based guidelines of surgical treatment for TCC patients. The general thinking has suggested that the larger extent of colon resection and lymph nodes examined, the more technical difficulties associated with surgical approaches and more chances to face the risk of postoperative morbidities [8]. However, due to the special tumor location and technique demanding for the surgical treatment of TCC, this group of patients is still presenting with severe disparities to make a selection between a less aggressive and extended surgical approach [9–11].

Laparoscopic surgery and open surgery have been found to be of equivalent value for patients with colon cancer. With the increased popularity in laparoscopic resection of TCC, both extended HC and less aggressive PC have been attempted [12]. Currently, none of study has evaluated the oncological outcomes between less aggressive PC and extended HC in TCC patients. This is the first large population-based study that investigated the oncological outcomes of PC compared with HC for TCC patients by matched-pair analysis. Here, this finding showed that PC performed for TCC is oncologically similar to HC with no significant difference in the rate of node positivity, and PC could not decrease the long-term survival benefit compared with extended HC. However, subgroup analyses indicated that, for patients with tumor size ≥ 5 cm, the 5-year CSS was prolonged among patients who underwent extended resection.

The main purpose of colon cancer surgery included the resection of primary tumor, an adequate margin as well as sufficient scope of lymphatic drainage. Hence, the type of surgical approach and the extent of lymphadenectomy were mainly based on tumor location. Tumors located at transverse colon could expand to regional lymph nodes along the middle colic, right colic and left colic vessels. The origin of the middle colon artery is considered as the scope of affected lymph nodes in TCC. This tumor location can be divided into right and left parts, which is based on the extent of lymph nodes along with the marginal artery in the middle colic artery [11]. Previous study has revealed the lymph node metastasis patterns in colon cancers. They reported that 7.6% to 11.1% of lymph nodes metastases through the right colic artery, but none of lymph nodes metastases along the ileocolic artery. In addition, lymph node metastasis has been reported to be confined to the middle colon artery if the tumor is located at the left side of middle colon artery, but metastases are detected along the right colon artery in 17% of patients with tumors on the right side of middle colon artery [13].

Previous study has implied that the surgeon can modify the surgical procedure to excise more tissue or use adjuvant techniques to aid the examination of lymph nodes by the pathologist [14]. In this study, we found that the lymph node count was far higher in TCC patients treated with HC than in those who treated with PC, but the number of nodes in the latter group was enough for proper staging. Moreover, other studies have showed that there were no differences in the recurrence rate between HC and PC [11]. Therefore, it is reasonable that removal of lymph nodes along the right colic artery may not affect the prognosis of TCC patients. Current studies have revealed that there were no metastatic lymph nodes along the ileocolic artery; it hence seems acceptable in TCC patients to undergo the less aggressive surgery with the ileocecal valve preserved [11]. To evaluate the oncologic safety, it is essential to understand both what is included in the unnecessary resection range beyond the actual resection required, and the number of positive lymph nodes examined, rather than only the number of lymph nodes examined. Removal of metastatic lymph nodes was necessary to stage accurately, which further influence on prognosis of TCC patients. However, extended resection for TCC patients could not lead to more proper staging compared with PC. To make an appropriate decision between HC and PC for TCC patients is mostly based on these evidences.

In clinical practice, the selection between PC and HC is not only determined by the oncological outcomes, there are also many other influence factors playing essential roles in determining the surgical approach of PC, such as emergency surgery, poor physical conditions, intestinal obstruction, unresectable distant metastasis and elderly patients with severe concomitant disease. In these cases, it is usually difficult for these patients to bear the prolonged anesthesia, expanded surgical strikes and higher risk of postoperative complication. Therefore, the less aggressive resection, PC, may also become a preferred option for these patients with TCC.

Strengths of this work included a more representative population of TCC patients, the large sample size and PSM analysis, which could provide more sufficient statistical power to greater generalizability of results in this study. Actually, in the consideration of HC or PC, it is also essential to understand the technical difficulties, postoperative complications, surgical approach (laparoscopy surgery or open surgery) and short-term outcomes, such as operation time, blood loss, time to fist flatus, time to liquid diet and postoperative stay. However, the SEER database lack the information mentioned above [15]. Despite of these limitations, the SEER database remains a valuable resource to assess the trends and patterns in patient characteristics, tumor features, cancer treatment and long-term survival outcomes [16].

In summary, this population-based study demonstrated that although PC contained smaller resection range and less lymph node examined than extended HC, the rate of node positivity under PC could not be accordingly decreased, and the long-term survival of patients treated with PC could not be reduced compared with extended resection. Hence, this study might indicate that the oncological outcomes of less aggressive PC were considered to be acceptable; this surgical approach is feasible and safe for selected TCC patients. However, the high quality study is still being needed to further confirm the findings of this work.

## MATERIALS AND METHODS

### Data source

We identified the cancer cases from the Surveillance, Epidemiology, and End-Results (SEER) cancer registry [17]. The SEER database includes the demographic, incidence and survival data from 17 population-based cancer registries, which covers about 28% of the US population. Population data and cancer cases could be extracted from SEER database [18]. All information in the SEER were anonymized and de-identified prior to release, which do not need informed consent from patients [16]. We have got permission to obtain research information file in the SEER program by National Cancer Institute, USA and the reference number was 10249-Nov2015. The study design was approved by the Ethics Committee of Cancer Hospital, Chinese Academy of Medical Sciences and Peking Union Medical College.

### Study population

We acquired patients diagnosed with TCC in stage I to III according to Site Recode classification. The collected patients were diagnosed from 2004 to 2013, because the seventh edition of AJCC stage system was available in SEER database since 2004. The surgical treatment for TCC patients included two approaches. 1) HC or greater (but less than total), right or left colectomy. The HC here is the resection of total right or left colon and a portion of transverse colon; 2) PC, partial removal of transverse colon and flexures. Other clinical characteristics extracted from the SEER database included gender, age, race, AJCC stage, tumor grade, histology and tumor size. The exclusion criteria include patients dead due to other causes and alive with no survival time.

### Statistical analysis

Firstly, we compared differences in patient characteristics between HC and PC group using the χ^2^ test. The cancer specific survival (CSS) was defined as the time from the TCC diagnosis until cancer recurrence or metastasis, cancer-related death and the end of follow up. The CSS was estimated with Kaplan-Meier method, and log-rank tests were used to compare the differences of CSS curves. Univariate and multivariate Cox's regression model were used to calculate hazard rate (HR) and exact 95% confidence intervals (CIs). Furthermore, the TCC patients were divided into 17 subgroups based on different patient and tumor characteristics including age, gender, AJCC stage, grade, histology and tumor size. Then subgroup analyses of CSS were separately performed using Cox regression model to observe prognostic consistency between HC and PC group. All statistical tests were two sided, *P* < 0.05 was considered to be statistical significance. The statistical analyses were evaluated by the statistical software package SPSS 20.0 (IBM Corp, Armonk, NY, USA) and R version 2.12.0 (www.r-project.org).

### PSM analysis

A propensity of 1:1 matched analysis was performed to reduce possible bias to minimum. Propensity scores were calculated using logistic regression model for each patient in both HC and PC group. The covariates included in the PSM analysis were gender, age, race, AJCC stage, AJCC T stage, AJCC N stage, grade, histology and tumor size. Covariates balance was evaluated between two groups by χ^2^ test. Patients in HC and PC group were well matched based on propensity score. The survival comparisons were then carried out in these matched TCC patients with the same methods as those in primary analysis.
